# In Vivo and in Vitro Regulatory Effect of Silibinin on Some Metabolic Enzyme Activities against Cobalt Induced Toxicity in Rats: A Biochemical Approach

**DOI:** 10.1021/acsomega.3c06953

**Published:** 2023-11-23

**Authors:** Yusuf Temel, H. Turan Akkoyun, Mahire Bayramoğlu Akkoyun, Fatma Karagözoğlu, Şule Melek, A. Ş̈ükrü Bengü, Sinem Aslan Erdem, Mehmet Çİftcí

**Affiliations:** † Solhan Health Services Vocational School, Bingol University, Bingol 12000, Turkey; ‡ Faculty of Veterinary Science, Department of Physiology, Siirt University, 56100 Siirt, Turkey; § Faculty of Veterinary Science, Department of Biochemistry, Siirt University, Siirt 56100, Turkey; ∥ Faculty of Veterinary Science, Department of Animal Nutrition and Nutritional Diseases, Dokuz Eylül University, İzmir 35890, Turkey; ⊥ Department of Surgery, Faculty of Veterinary Science, Bingol Universıty, Bingöl 12000, Turkey; # Vocational School of Health Services, Bingöl University, Bingöl 12000, Turkey; ¶ Department of Pharmacognosy, Faculty of Pharmacy, Ankara University, Ankara 06560, Turkey; ∇ Faculty of Veterinary Science, Department of Biochemistry, Bingöl University, Bingöl 12000, Turkey

## Abstract

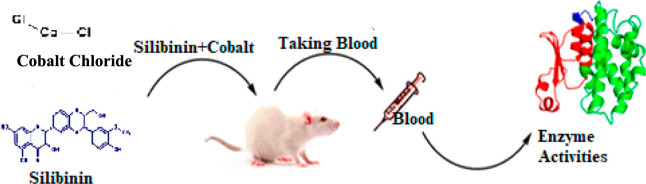

The study aimed to examine the in vivo inhibition effect of cobalt ion and silibinin on metabolic enzymes such as glucose-6-phosphate dehydrogenase (G6PD), 6-phosphogluconate dehydrogenase (6PGD), glutathione reductase (GR), and glutathione S-transferase (GST) and their in vitro inhibition effect on 6PGD. Twenty-four Wistar Albino rats weighing approximately 250–300 g were used in the study. The rats were divided into 4 groups as group 1 (control): isotonic serum (0.5 mL i.p), group 2 (cobalt): (150 mg kg/day cobalt), group 3 (silibinin): (100 mg/kg/day silibinin), group 4 (cobalt + silibinin). As a result of the in vivo applications, a statistically significant decrease was observed in the activities of G6PD (*p* < 0.05), 6PGD (*p* < 0.05), GR (*p* < 0.05), and GST (*p* < 0.05) enzymes in the groups that were administered cobalt compared to the control group. It was also found that the activities of G6PD (*p* < 0.05), 6PGD (*p* > 0.05), GR (*p* > 0.05), and GST (*p* > 0.05) enzymes increased in groups that were administered cobalt + silibinin compared to the group that was administered cobalt. As for in vitro applications, it was found that different Co^2+^ ions inhibited 6PGD enzyme which was obtained as a result of purification with IC_50_ = 346.6 μM value, while silibinin increased 6PGD enzyme activity within the concentration range of 100–750 μM by 40%. As a result, it was found that cobalt ions had an inhibition effect on G6PD, GR, and GST enzymes, which are vitally important for living metabolism, in vitro and in vivo and inhibited 6PGD enzyme activity in vitro, and silibinin increased these enzyme activities in vivo and 6PGD enzyme activity both in vivo and in vitro and decreased the inhibition effect.

## 1 Introduction

It has been stated that trace elements play a significant role in the metabolic process and functioning of many enzymatic systems by providing biochemical modulation in many living things, including the human body.^1−3^ It has been reported that deficiency in physiological levels of trace elements leads to a decrease in vulnerability against the unstable antioxidant defense and oxidative stress,^4^ and that overconcentration of certain trace elements could be among the reasons for elevated free radical development and lipid peroxidation.^5^ Cobalt (Co) is a precious metal that is utilized in producing metal alloys, batteries, and pigments.^6^ Co is essential for human health, as it is a component of the B12 vitamin complex that plays a role in the production of red blood cells and regulation of DNA synthesis, fatty acids, and amino acid metabolisms.^7^ However, high concentration of cobalt is toxic for humans, land and sea animals, and plants.^8^ Many researchers have reported that acute and chronic cobalt toxicity harms various organs.^9^ Professional exposure to cobalt element happens mainly through respiration in industries related to the manufacture, processing, and use of metal and in factories that produce hard metals, and it is associated with the development of interstitial lung disease named as hard metal lung disease.^10−12^ Silibinin, which is the main active component of the silymarin complex, is usually obtained from the milk thistle. Silibinin, which has a wide area of use in order to protect the liver, kidneys, and heart tissue, has an antioxidant property.^13,14^ Recent studies have been oriented toward the anticancer effect of silibinin, and it has been shown that it could be used as a protective and therapeutic agent in cancer treatment.^15^ G6PD is the primary and speed-limiting enzyme of the pentose phosphate pathway that yields the production of ribose-5-phosphate and NADPH (β-nicotinamide adenine dinucleotide 2′-phosphate reduced).^16^ NADPH coenzyme is included in the synthesis of certain amino acids, protects the cells from oxidants, and plays a role in the detoxification of xenobiotics by way of the glutathione reductase-peroxidase system.^17^ 6PGD (E.C.1.1.1.44) is the third enzyme of the pentose phosphate metabolic pathway, and in the presence of NADP^+^, it behaves as a catalyzer in the conversion of 6-PGA (6-phosphogluconate) into d-ribulose-5-phosphate.^18,19^ NADPH protects the cell against oxidant agents by producing reduced glutathione (GSH).^20^ One of the important cellular antioxidant enzymes is GR, and it catalyzes the conversion of glutathione from its oxidized form into its reduced form.^21,22^ GSTs are multifunctional enzymes found everywhere and represent 10% of cytosolic proteins. They generate the conjugation of toxic xenobiotics and compounds that are produced oxidatively. Thus, they enable the elimination of metabolisms and provide protection from oxidants.^23^ In the study, it was aimed to examine the inhibition effects of the cobalt ion on G6PD, 6PGD, GR and GST enzymes and to determine whether silibinin prevents the side effects of the cobalt ion in vivo and in vitro.

## 2 Materials and Methods

### 2.1 Chemicals

Silibinin was obtained from Sigma Chemical Co. (St. Louis, USA), and cobalt was procured from Sigma-Aldrich. G6PD, 6PGD, Tris, NADP^+^, protein assay reagent, NADPH, DTNB, and standard serum albumin were purchased from Pharmacia (New Jersey, USA), and 2′,5′-ADP-Sepharose 4B was provided by Sigma-Aldrich (St. Louis, MO and Darmstadt, Germany). All other chemicals were obtained from Merck or Sigma (Germany) with analytical purity.

### 2.2 In Vivo Effect of Silibinin and Cobalt

Ethical approval for the study was obtained from the Bingol University Animal Experiments Ethics Board (BUHADEK:18.05.2021-2021/02). All experiments were conducted in line with the ethical rules included in the Laboratory Animals Care and Use Guidelines. Twenty-four Wistar Albino rats that were 12 weeks old and weighed approximately 250–300 g were used in the study. The rats were divided into four groups. They were fed with standard feed (commercial food pellets; crude protein 24%, phosphorus 0.52%, crude fat 4%, sodium 0.17%, crude cellulose 6.28%, crude ash 7.06%, calcium 1.00%), and they were allowed to drink water. The environmental temperature was kept constant at 20–25 °C for the rats. Normal lighting and darkness (12L:12D) were arranged as a constant photoperiod. Group 1 (control) was administered isotonic serum (0.5 mL, i.p), group 2 (cobalt) (150 mg/kg/day/cobalt) (oral),^24^ group 3 (silibinin): 100 mg/kg/day silibinin (oral),^25^ and group 4 (cobalt + silibinin): (150 mg/kg/day-cobalt) **+** 100 mg/kg/day-silibinin (oral gavage). The animals fasted overnight (for about 12) on the last day of the experimental model before the induction of anesthesia or the collection of blood samples. At the end of day 7, the rats were anesthetized with (60 mg/kg ip) ketamine hydrochloride and 10 mg/kg Xylazine i.p. Kidney tissue was extracted following median laparotomy and was washed in phosphate-buffered saline and kept in deep freeze (−80 °C) until analysis time.

### 2.3 Preparation of Supernatant from Tissue Samples of Kidney

Tris-HCl tampon of 20 mM at pH 7.4 was added to 200 mg of kidney tissue, and the mixture was homogenized by using a homogenizer device (Ultra Turrax-T25). Then, it was centrifuged at 15,000 rpm at 4 °C for 30 min. The supernatant on the surface was moved to a new tube.^26^

### 2.4 Purification of 6PGD Enzyme

In line with the method determined by Temel et al. (2017), 2′,5′-ADP-Sepharose 4B column was prepared.^27,28^ The prepared supernatant was applied to the colon, along with 10 mL of the colon material. After that, the colon was washed by using 50 mM phosphate tampon with pH = 7.35. 6PGD was washed by using 80 mM phosphate + 0.5 mM 1 mMEDTA + NAPD tampon with pH = 7.35. All applications were performed at 4 °C.

### 2.5 Measurement of Activity of Some Metabolic Enzymes

In vivo activities of G6PD and 6PGD enzymes and in vitro activity of the 6PGD enzyme were measured according to the Beutler method.^29^ Activity of the GR enzyme was measured by using the Carlberg and Mannervik method.^30^ Habig method was employed in measuring the activity of GST enzyme. Enzyme activities were expressed as U/mgprot.^31^

### 2.6 Determination of Protein Quantity

Kidney tissue protein levels were made spectrophotometrically at 595 nm according to the Bradford method and it was formed by using standard graphic bovine serum albumin.^32^

### 2.7 In Vitro Effect of Silibinin and Cobalt

In order to evaluate the effect of silibinin and cobalt on the activity of 6PGD enzyme, five different concentrations of silibinin (100, 200, 400, 500, and 750 μM) and five different concentrations of cobalt (38.5, 77, 192.5, 385, and 770 μM) were separately placed in tubes that contained purified enzyme. IC_50_ values (inhibitor concentration that decreases the total enzyme activity by 50%) were determined over % activity – [*I*] graphs.^33^

### 2.8 Analysis of Kinetic Data

The study data obtained were presented as mean ± standard deviation. The differences were defined as significant at *p* < 0.05. Shapiro–Wilk was done to evaluate the normality of the data, and Levene’s tests were performed to evaluate the homogeneity of the data. In vivo effects of cobalt and silibinin on rat kidney G6PD, 6PGD, GR, and GST enzyme activity of groups were analyzed by one-way analysis of variance (ANOVA) followed by Tukey’s multiple comparisons test. For statistical analyses, IBM SPSS Statistics for Windows, version 22.0 (IBM Corp., N.Y., USA) and GraphPad Prism for Windows ver. 5.0 program (San Diego, CA, USA) were employed.

## 3 Results

**Figure 1:**
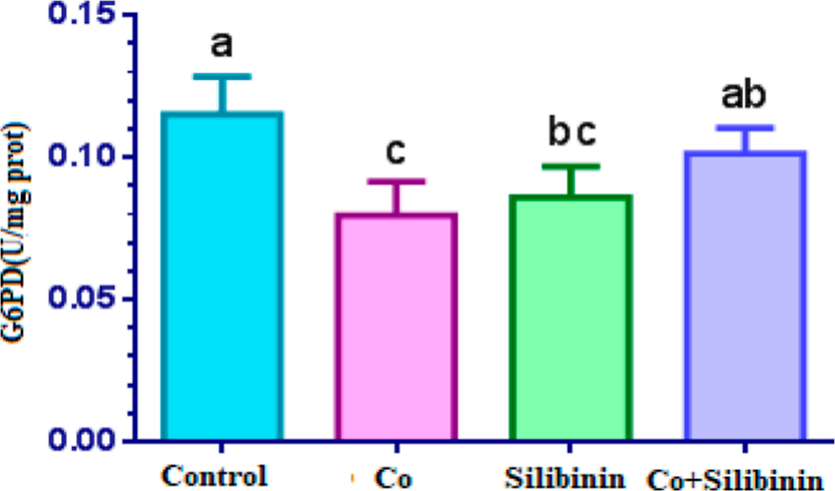
Effects of cobalt and silibinin on the activity of the G6PD enzyme in rat kidney in vivo. Different letters in (a–c) represent statistical differences between the groups (*p* < 0.05).

With respect to the control group, a statistically significant decrease in G6PD enzyme activity was observed in the cobalt group (*p* < 0.05). While a higher G6PD enzyme activity was found in the silibinin group compared to the cobalt group, G6PD enzyme activity in the silibinin group was determined to be statistically significant lower in comparison to the control group (*p* < 0.05). In the cobalt + silibinin group, G6PD enzyme activity displayed a statistically significant increase with respect to the cobalt group (*p* < 0.05), and G6PD enzyme activity in this group approximated the control group (*p* > 0.05) (Figure 1).

**Figure 2:**
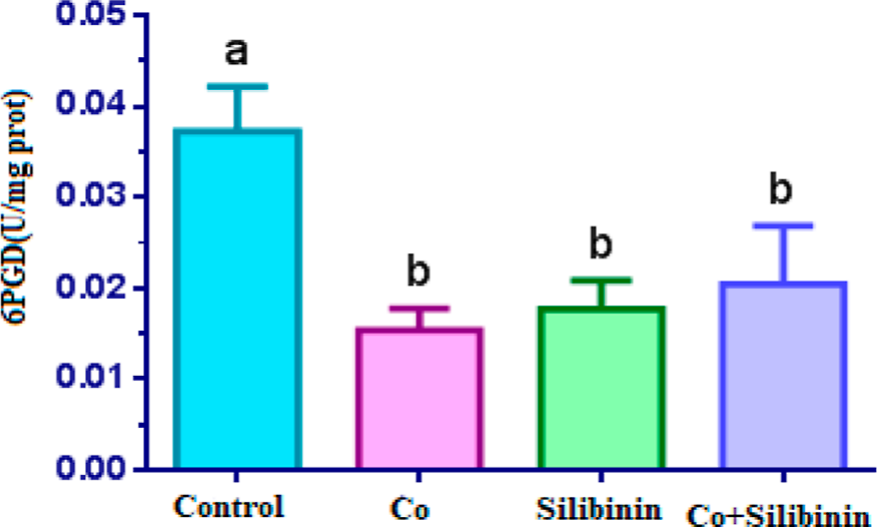
Effects of cobalt and silibinin on the activity of the 6PGD enzyme in rat kidney in vivo. Different letters in (a–c) represent statistical differences between the groups (*p* < 0.05).

When compared with the control group, a statistically significant decrease in 6PGD enzyme activity was found in the cobalt group (*p* < 0.05). 6PGD enzyme activity was higher in the silibinin group in comparison to the cobalt group (*p* < 0.05), but the activity of this enzyme in the silibinin group was statistically significantly lower compared to the control group (*p* < 0.05). As for the cobalt + silibinin group, 6PGD enzyme activity was statistically insignificantly higher in comparison to the control group (*p* > 0.05), while a statistically significantly decrease was seen in 6PGD enzyme activity in this group with respect to the control group (*p* < 0.05) (Figure 2).

**Figure 3:**
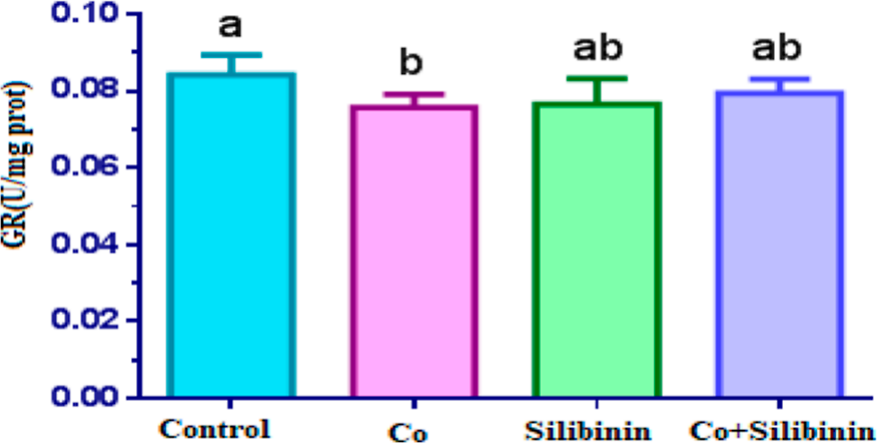
Effects of cobalt and silibinin on the activity of the GR enzyme in rat kidney in vivo. Different letters in (a–c) represent statistical differences between the groups (*p* < 0.05).

In comparison to the control group, a statistically significant decrease in GR enzyme activity was found in the cobalt group (*p* < 0.05). The comparison of the silibinin group with the cobalt group showed a higher GR enzyme activity in the silibinin group (*p* > 0.05), but GR enzyme activity in the silibinin group was statistically insignificantly lower when compared with the control group (*p* < 0.05). Regarding the cobalt + silibinin group, GR enzyme activity in this group was found to be statistically insignificantly higher compared to the cobalt group (*p* < 0.05). GR enzyme activity in the cobalt + silibinin group increased in comparison to the cobalt group and approximated the control group (*p* > 0.05) (Figure 3).

**Figure 4:**
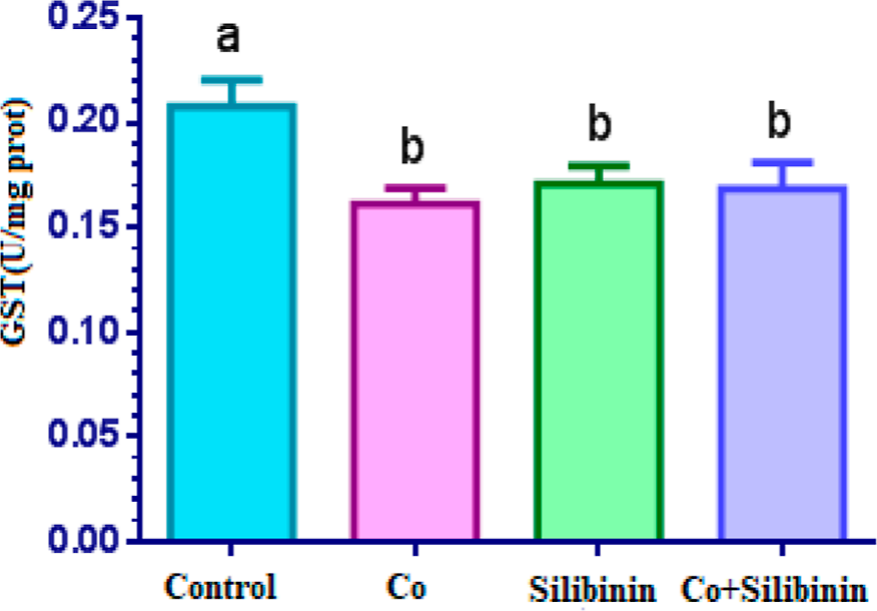
Effects of cobalt and silibinin on the activity of the GR enzyme in rat kidney in vivo. Different letters in (a–c) represent statistical differences between the groups (*p* < 0.05).

A statistically significant decrease in GST enzyme activity was observed in the cobalt group in comparison to the control group (*p* < 0.05). When the silibinin group was compared against the cobalt group, GST enzyme activity was found to be higher in the silibinin group (*p* > 0.05), but when compared with the control group, GST enzyme activity in the silibinin group was determined to be statistically significantly lower (*p* < 0.05). GST enzyme activity in the cobalt-silibinin group was determined to be statistically significantly lower in comparison to the control group (*p* < 0.05), while it increased with respect to the cobalt group (*p* > 0.05) (Figure 4).

**Figure 5:**
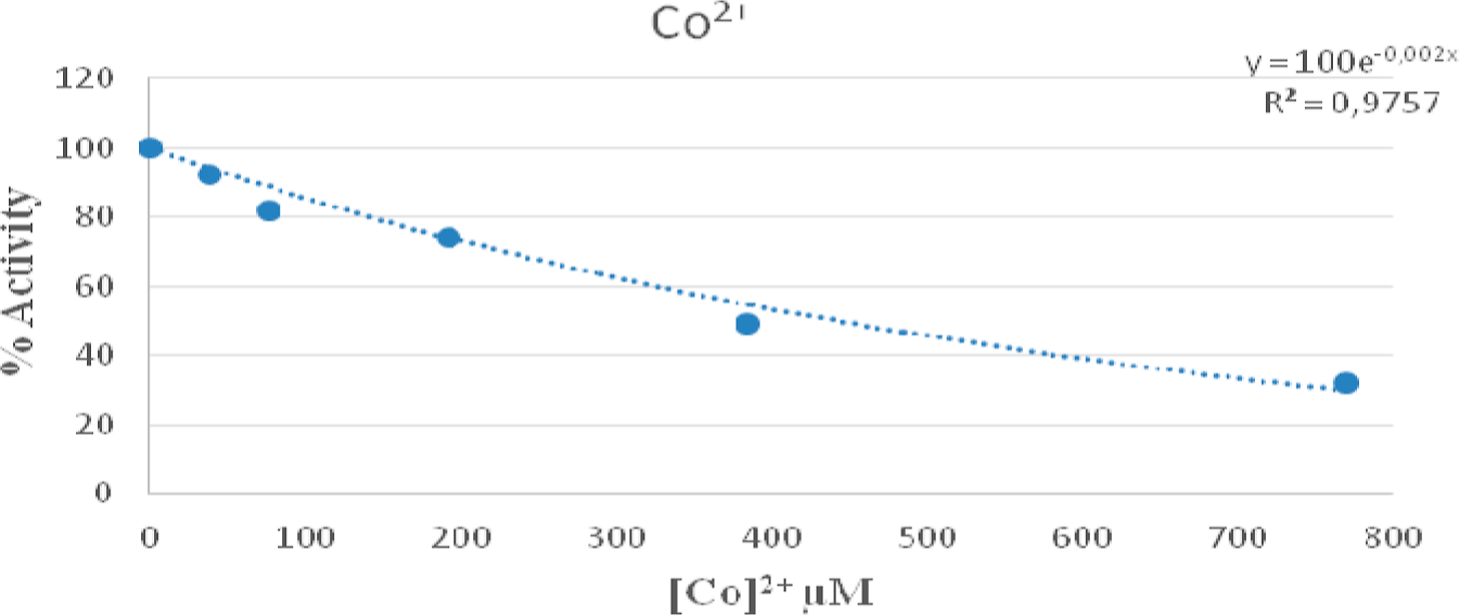
In vitro effect of cobalt ions on the rat kidney 6PGD enzyme activity.

**Figure 6:**
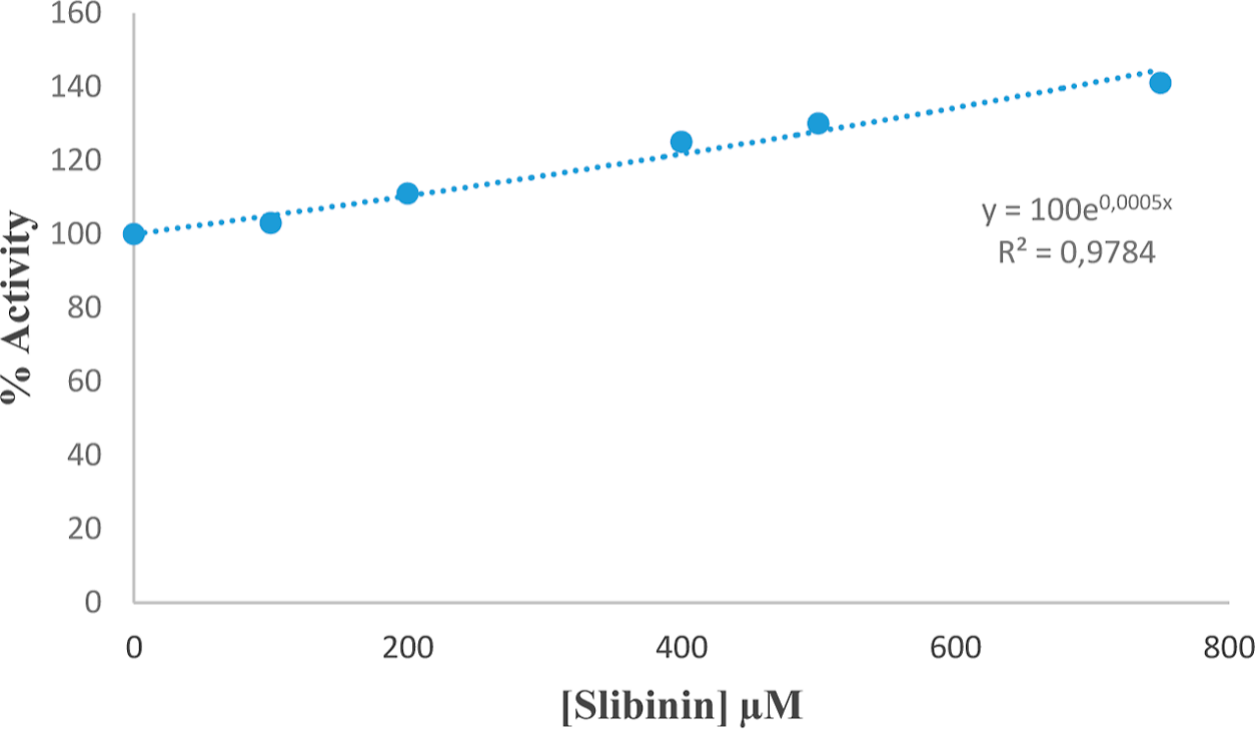
In vitro effect of silibinin on rat kidney 6PGD enzyme activity.

In *in vitro* applications, 6PGD enzyme was obtained from rat kidney tissues by using 2′,5′-ADP-sefaroz-4B affinity chromatography. The effect of Co^2+^ in different concentrations on 6PGD enzyme activity, which was obtained in pure form, was examined through the spectrophotometric method. When the data obtained were analyzed, it was found that Co^2+^ ions inhibited the enzyme at the value of IC_50_ = 346.6 μM. It was also determined that silibinin increased 6PGD enzyme activity by 40% within the concentration range of 100–750 μM (Figures 5 and 6).

## 4 Discussion

Human beings are exposed to complicated toxic compound mixtures in their homes and professional environments. These chemical compounds create negative effects on aquatic biota, animals, and humans through water reservoirs, water courses, and rivers.^34^ As cobalt is utilized as a pigment in glass, ceramics, and paints and cobalt alloys are intensely employed in aircraft engines, magnets, and artificial connections, environmental exposure can occur at high levels, particularly in industrial environments. Workers employed in metal mining, melting, and refining can get exposed to higher amounts of cobalt.^35,36^ Being exposed to high levels of soluble cobalt salts are toxic, and the median varying between 150 and 500 mg kg^–1^ has been reported to be lethal dose in mammals.^37^ Cobalt in high doses, which is a cofactor for the activation of various enzymes and formation of B12 vitamin and other cobalamins, creates various effects on the heart, thyroids, liver, and kidneys.^34^ Cobalt, which is among the important heavy metals that contribute to the production of free oxygen radicals, is known to be a highly toxic metal. Moreover, it has been reported in some papers that it is an important free radical H_2_O_2_ generator.^38^ The cellular condition in which the generation of reactive oxygen species exceeds antioxidant capacity is defined as oxidative stress. It has been reported to significantly contribute to the pathogenesis of many human and animal diseases, including cardiovascular and renal disorders.^39^ The role played by oxidative stress in cobalt-induced organ pathologies have frequently been expressed in studies conducted.^8^

In the present study, the purpose was to investigate in vivo inhibition effects of cobalt ion and silibinin on metabolic enzymes such as G6PD, 6PGD, GR, and GST and their in vitro inhibition effects on 6PGD enzyme. When the study results were analyzed, it was seen that G6PD enzyme activity statistically significantly decreased in the cobalt group in comparison to the control group (*p* < 0.05). It was determined that the activity of the enzyme statistically significantly rose in the cobalt + silibinin group when compared to the cobalt group (*p* < 0.05), Regarding 6PGD enzyme activity, while a statistically significant drop in 6PGD enzyme activity was observed in the cobalt group with respect to the control group (*p* < 0.05), the enzyme activity in the silibinin group was found to be higher in the silibinin group compared to the cobalt group, but the increase was not statistically significant. 6PGD enzyme activity in the cobalt + silibinin group statistically insignificantly increased in comparison to the cobalt group (*p* > 0.05). As regards GR and GST enzyme activities, a statistically significant decrease was observed in the enzyme activity in the cobalt group compared to the control group (*p* < 0.05), while an increase in the enzyme activities was found in the cobalt + silibinin group with respect to the cobalt group. GST enzyme activity in the cobalt + silibinin group was statistically significantly lower compared to the control group (*p* < 0.05). When the effects of cobalt ion on 6PGD enzyme, which was obtained from the rat kidney tissue through purification by using 2′,5′ ADP-sepharose-4B affinity chromatography, were evaluated, it was determined that Co^2+^ ions restricted the enzyme at the value of IC_50_ = 346.6 μM, and that silibinin increased 6PGD enzyme activity by 40% at the concentration range of 100–750 μM.

In different studies conducted, it was reported that exposure to CoCl_2_ in Wistar rats led to significant increases in oxidative stress parameters (hydrogen peroxide, H_2_O_2_, and malondialdehyde, MDA), as well as causing a decrease in superoxide dismutase (SOD) activity in reduced glutathione (GSH) in kidneys and adaptive increases in GST and catalase (CAT).^40^ It was determined that high-dose cobalt application in rats considerably increased the concentrations of AST, ALT, and CK enzymes, and that no significant effect on the kidneys was observed in the pathological evaluation.^41^ In another study conducted, it was reported that cobalt amassed the most in the kidney, followed by the liver, blood, and lungs, in descending order and depending on the dose. Assessment of the liver and kidney function tests showed a sharp increase in serum urea and creatinine levels depending on the dose, and it was reported that the compound led to a higher degree of toxicity in the kidney compared to the liver.^42^ It was determined in a study that goldfish (*Carassius auratus*) that was exposed to Co^2+^ at 50, 100, or 150 mg L^–1^ concentrations for 96 h, protein carbonyl content in the kidney increased, that catalase activity did not change, that superoxide dismutase enzyme activity increased, that glutathione peroxidase and glutathione S-transferase activities did not change, but that glutathione reductase activity increased by 70%.^8^ Silymarin and its main component, silibinin, are extracted from the healing plant *Silybum marianum* (milk thistle), and they have conventionally been used in treating diseases. In recent times, it has been shown that active flavonoid agents displayed considerable antineoplastic effects in various in vitro and in vivo cancer models, such as colon, breast, skin, bladder, prostate, and kidney carcinomas.^43^ In various studies conducted, it was reported that silibinin was effective in treating arsenic-induced nephrotoxicity by eliminating oxidative stress, inflammation, and apoptosis in rats, and that silibinin also activated GST enzyme activity.^44^ In the study conducted by Yassin et al. (2021), in which they examined the effectiveness of silymarin and silibinin against experimentally induced renal carcinogenesis in male Wistar rats, it was reported that silymarin and silibinin application weakened toxicity indicators in serum, decreased lipid peroxidation, considerably strengthened renal antioxidant arsenal, as well as increased GST enzyme activity and decreased GR enzyme activity.^45^ In vitro experiments showed that silibinin nanoparticles protect liver cells and reduce cellular damage. This was shown by decreased serum ALP, ALT, and AST levels.^46^ In a different study, it was reported that doxorubicin treatment caused damage to the heart, but the coadministration of silymarin/silibinin protected the heart from this damage.^47^ In another study, it was reported that silibinin could have significant positive effects against nephrotoxicity.^48^ It was also reported that silibinin considerably reduced morphological changes observed in the S3 segment of the proximal tubule in the kidneys.^49^ It was determined that the results of the studies mentioned above were consistent with the results of the study and supported it.

## 5 Conclusions

It has been stated in the literature that cobalt and its alloys have a wide range of use in industrial fields, that higher levels of environmental exposure can occur, especially in industrialized countries, and that exposure to high levels of soluble cobalt salts creates a toxic effect in living things. In this study, we found that cobalt ions strongly inhibited G6PD, GR, and GST enzymes in vivo and inhibited 6PGD enzyme activity in vivo and in vitro. We also determined that silibinin, whose antioxidant properties have been demonstrated in previous studies, reduced the inhibitory effect of cobalt ions on these enzymes. These enzymes are important for antioxidant metabolism and are also associated with the formation of various types of cancer. For these reasons, we anticipate that the results of this study will shed light on the pathophysiology of diseases caused by cobalt toxicity and that silibinin may be a potential phytotherapy approach for the treatment of cobalt toxicity.

## Data Availability

All data generated or analyzed during this study are included in this published article.

## Abbreviations

DTNB: 5,5-dithiobis (2-nitrobenzoicacid)
DDT: dichlorodi-phenyltrichloroethane
CDNB: 1-chloro-2,4-dinitrobenzene
EDTA: thylenediaminetetra acetic acid
G6PD: glucose6-phosphatedehydrogenase
6PGD: 6-phosphogluconate dehydrogenase
GR: glutathione reductase
GST: glutathione S-transferase
Co: cobalt
NADP^+^: nicotinamide adenine dinucleotide phosphate
C_25_H_22_O_10_: silibinin
